# Recurrent adenoid cystic carcinoma of the vulva: A case report of salvage stereotactic ablative radiotherapy to the pudendal nerve

**DOI:** 10.1016/j.radcr.2025.06.069

**Published:** 2025-07-26

**Authors:** Hanya Irfan, Yusuf Ben-Tarifite, Rana Mahmood

**Affiliations:** a Whittington Hospital Trust, Magdala Avenue, London, N19 5NF, England; b Oxford University Hospitals Trust, Headley Way, Headington, Oxford, OX3 9DU, England; c Mediclinic City Hospital, Dubai Healthcare City, Bldg 37-26th St, Umm Hurair 2, Dubai, United Arab Emirates

**Keywords:** Adenoid cystic carcinoma, Bartholin’s glands, Carcinoma, Stereotactic ablative radiotherapy, Vulval cancer, Gynecological-oncology

## Abstract

Adenoid cystic carcinoma (ACC) is an indolent malignancy that frequently recurs and particularly involves surrounding nerves which complicates management. ACC is mostly reported in the head-and-neck, but can also arise in the pelvis where treatment strategies are less established due to its rarity. In this case report a 44-year-old patient with previously treated vulval ACC presented with an isolated recurrence along the dorsal branch of the pudendal nerve. The pudendal nerve was successfully treated with salvage stereotactic ablative radiotherapy (SABR), with no evidence of active disease at 1-year follow-up. There is a potential role for SABR as an effective treatment for recurrent ACC with perineural spread, particularly when surgical options are limited. This case highlights the necessity of individualized treatment planning in rare anatomical presentations of ACC, with consideration for balancing oncological control with aim to preserve function. This report contributes to the limited literature on vulvar ACC and supports the integration of adjuvant radiotherapy into the multidisciplinary management.

## Introduction

Adenoid cystic carcinoma (ACC) is a rare slow-growing cancer of the exocrine glands that frequently invades local structures including nerve pathways. ACC is most reported in the head-and-neck region. Therefore, predicting the path of perineural invasion in these instances is more established, for example treatment protocol for ACC of the parotid glands could involve radiotherapy to the facial nerve. However, it can also arise in other sites such as breast, lungs, prostate and cervix. When ACC occurs in rarer anatomical sites, like the vulva in this case, its clinical behavior and the optimal regarded management are less well-defined. Generally, surgical excision is the gold standard with consideration of adjuvant chemo-radiotherapy dependent on clinical judgement due to its high capacity for recurrence. In particular, the rarity of ACC in the vulva poses unique challenges, particularly in identifying potential patterns of recurrence leading to uncertainty regarding treatment strategies. With this background, this case report describes a rare presentation of ACC of the vulva with recurrence along the dorsal branch of the pudendal nerve, and successful salvage treatment using stereotactic ablative radiotherapy (SABR).

## Case presentation

**Fig. 1:**
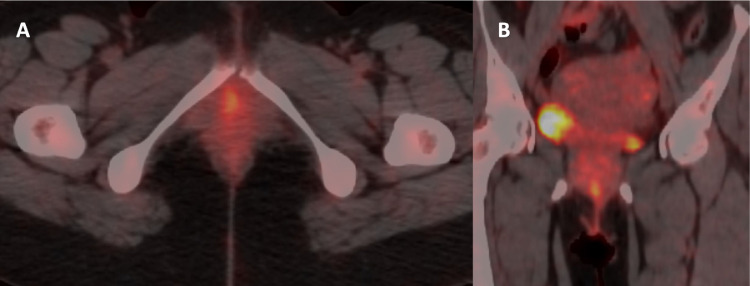
FDG PET CT scan of a 39-year-old female, presenting with rapidly enlarging 1 cm vulval lump, shows FDG-avid metabolically active lesion involving right vulva without invasion of the urethra, bone or muscle. June 2019, preoperative. (A, axial view; B, coronal view).

**Fig. 2:**
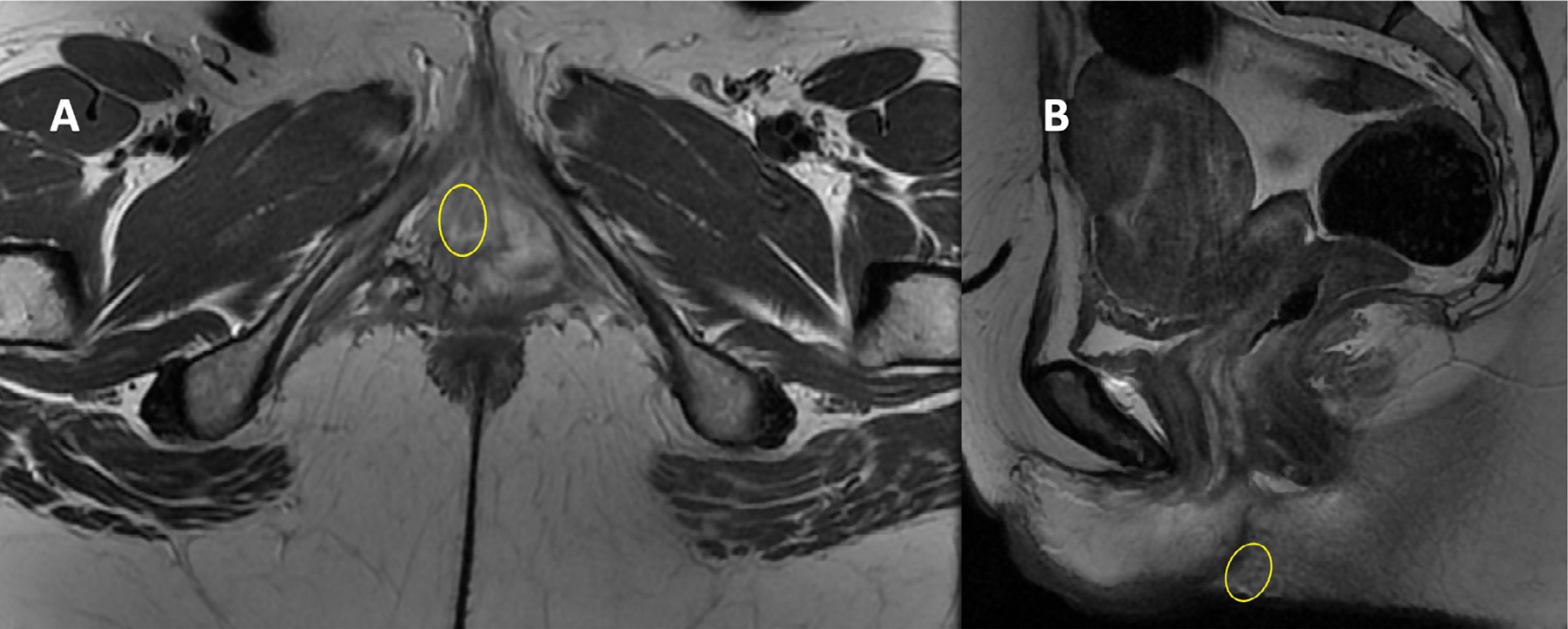
MRI T2W scan of a 39-year-old female, presenting with rapidly enlarging 1 cm vulval lump, shows FDG-avid lesion involving right vulva without invasion of the urethra, bone or muscle. June 2019, preoperative. (A, axial view; B, sagittal view).

In 2019 a 39-year-old female, gravida 2 para 2, presented with a 1 cm vulval lump. She first noticed it 2 months prior, but it was rapidly growing. There was no itching, bleeding, or pain. On clinical examination there was a solid mass, 1cm in diameter with normal overlying skin. There were no enlarged inguinal nodes. Per vaginal and speculum examination was normal. There was no deficit to local or perineal sensation. Biopsy confirmed this mass to be ACC, and preoperative imaging was done to stage the lesion and plan management as below (Fig. 1, Fig. 2).

Given the mass’s localized nature, wide-local excision (WLE) was undertaken with minimal residual tumor. There was no scope for further resection due to proximity to critical anatomical structures like the urethra. Therefore, the patient underwent adjuvant high-dose-rate (HDR) brachytherapy (5 fractions) delivered via a vaginal cylinder. Consequently, the patient suffered significant vaginal narrowing leading to long-standing sexual inactivity. Despite recommendations for using vaginal dilators to mitigate the radiotherapy side-effects, compliance was an issue. Routine follow up with MRI and PET imaging over the next 4 years showed no disease recurrence, and the patient remained asymptomatic.

**Fig. 3:**
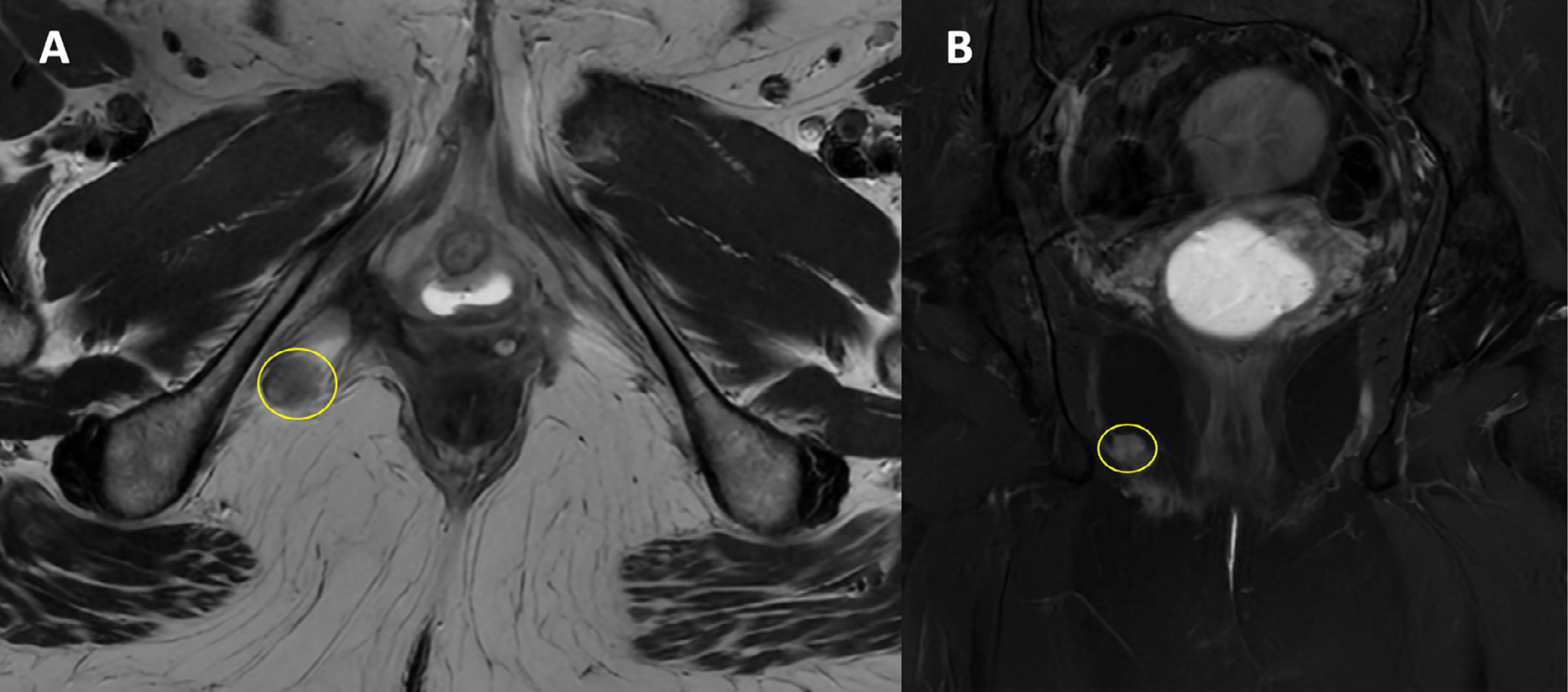
MRI scan T2w with gadolinium contrast, performed to assess for local recurrence, showed a 1 cm hyperintense lesion at the pudendal nerve route without invading the side wall, muscles and bones. May 2023, pre-SABR. (A, axial view T2w; B, coronal view, T2w Fat-suppressed).

**Fig. 4:**
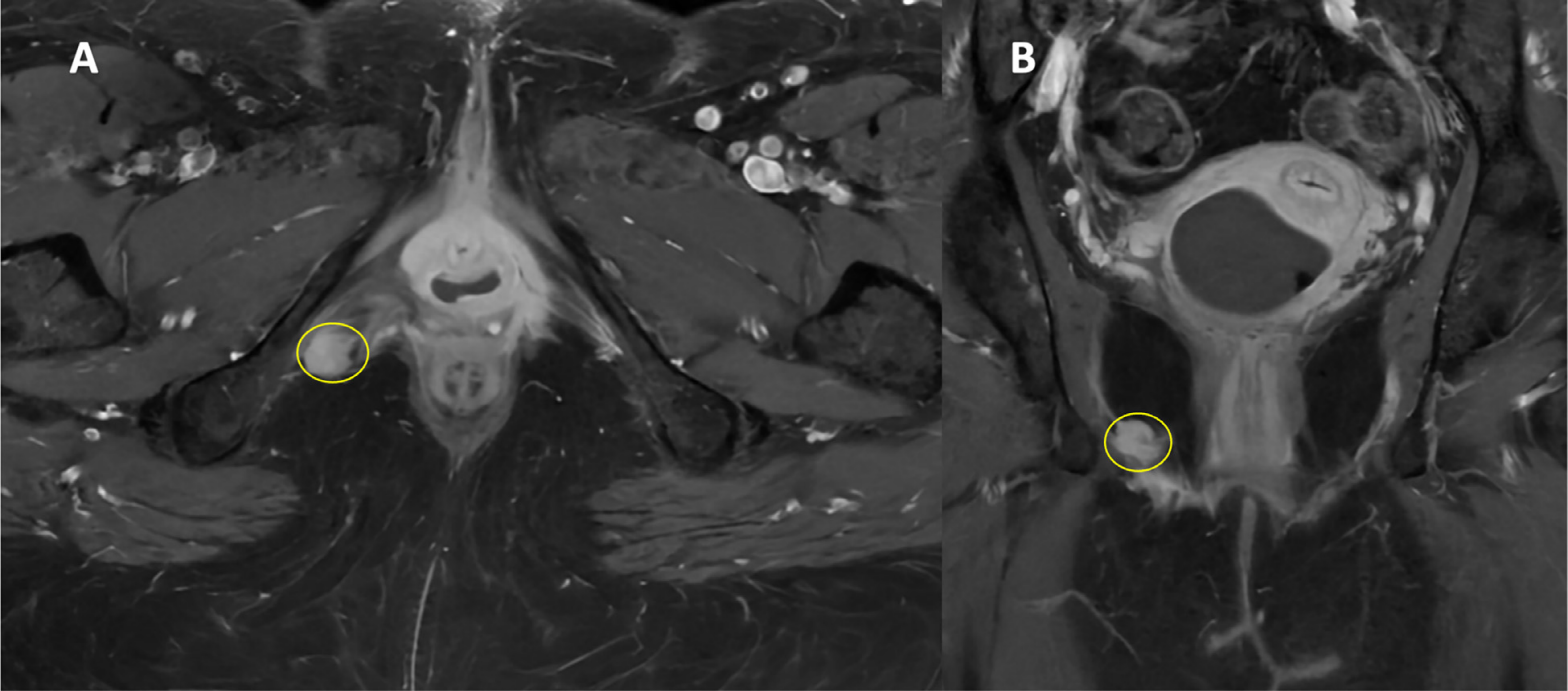
MRI scan T1w with gadolinium contrast, performed to assess for local recurrence, showed a 1 cm contrast enhancing hyperintense lesion at the pudendal nerve route without invading the side wall, muscles and bones. May 2023, pre-SABR. (A, axial view; B, coronal view).

In May 2023, routine follow-up imaging revealed a new 1 cm lesion in the right lower pelvis along the right inferior ischiopubic ramus (Fig. 3, Fig. 4).

Given the background and location, this lesion was suspicious for ACC recurrence along the dorsal branch of the pudendal nerve. A PET scan confirmed metabolic activity in this region, confirming the cancer recurrence, and no further distant metastases were identified. A biopsy was performed, given the unusual site and delayed recurrence, which confirmed the lesion as a recurrence of the ACC.

**Fig. 5:**
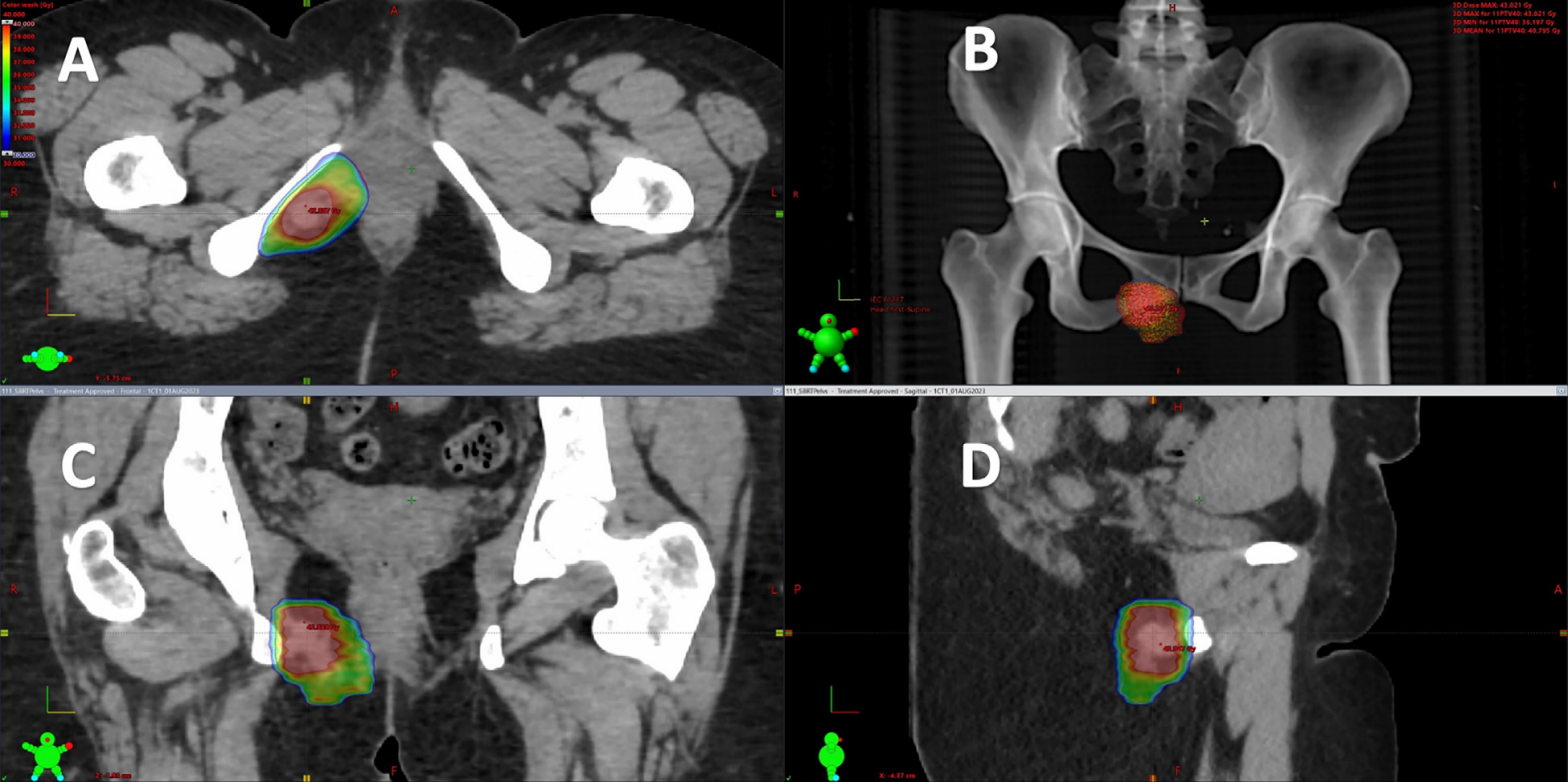
Radiotherapy plan shows stereotactic ablative radiotherapy (SABR) color wash (A, axial; B, digital reconstructed radiograph; C, coronal view; D, sagittal view). Radiotherapy software-generated 360 degrees images displayed in green Figure to orient the imaging plane.

**Fig. 6:**
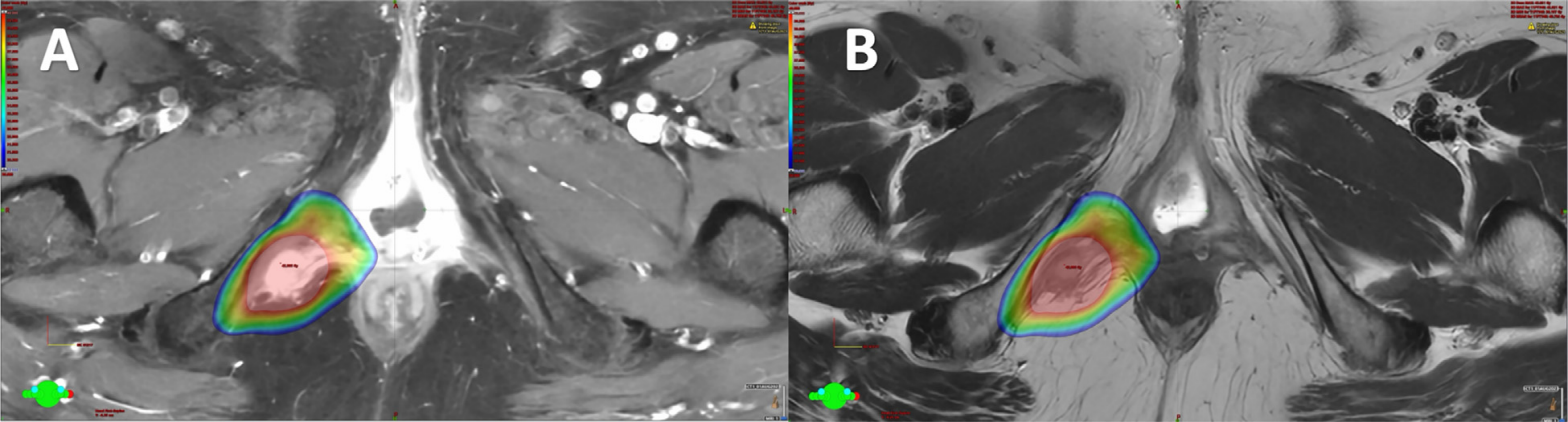
SABR dose displayed on co-registered MRI scan in August 2023 (A, T1w+C MRI scan; B, MRI T2w scan) Radiotherapy software-generated 360 degrees images displayed in green Fig. to orient the imaging plane.

This case was brought to the multidisciplinary team (MDT) meeting, with input from gynecological-oncology surgeons, oncologists, and radiology. The recommendation was made to pursue salvage stereotactic ablative radiotherapy (SABR), a high-precision radiation technique. The SABR treatment plan involved 5 sessions over 2 weeks, delivering a total dose of 40 Gy. The goal was to achieve local control of the tumor while minimizing radiation exposure to surrounding structures. Informed consent was obtained after full discussion of the aim and potential toxicity of SABR. These potential risks included pudendal nerve damage leading to perineal pain or altered sensation, and also a small risk of per vaginal or rectal bleeding. A CT planning scan with an empty rectum and a comfortably full bladder was acquired and later co-registered with MRI scan (T2 weighted and T1 with contrast). Total dose of 35 Gy to Planning (PTV) and 40 Gy to Gross tumor volume (GTV) was prescribed over 5 fractions and delivered on alternate days, as illustrated below (Fig. 5, Fig. 6). The patient tolerated radiotherapy without any toxicity.

**Fig. 7:**
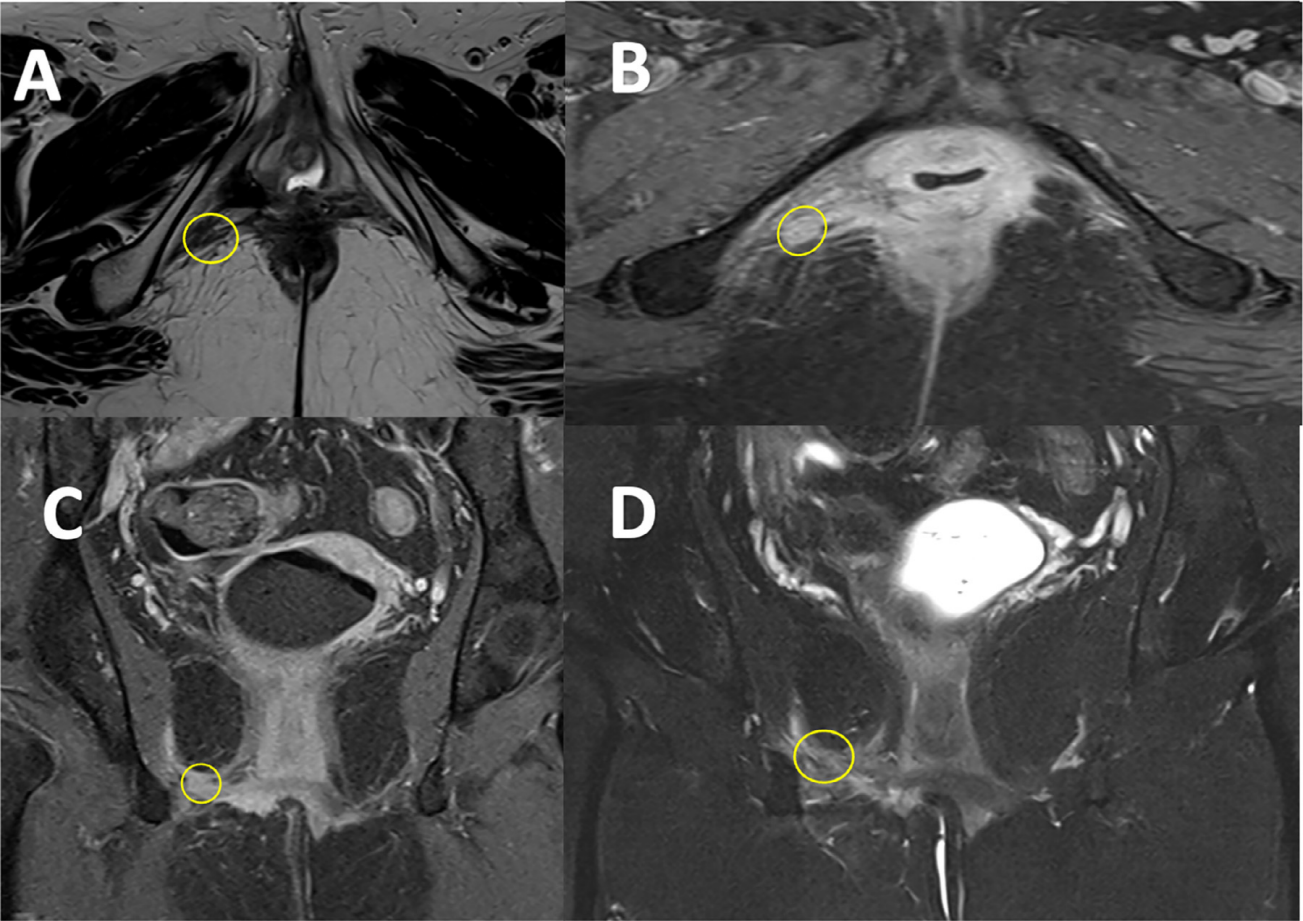
October 2023: MRI scan performed 3 months after SABR showed significant regression of the lesion, becoming isointense and no further contrast enhancement. Tumor Board review confirmed this to be nonviable fibrotic tissue (A, T2w axial; B, T1w axial with contrast; C, T1w coronal with contrast; D, T2w fat-suppressed coronal).

**Fig. 8:**
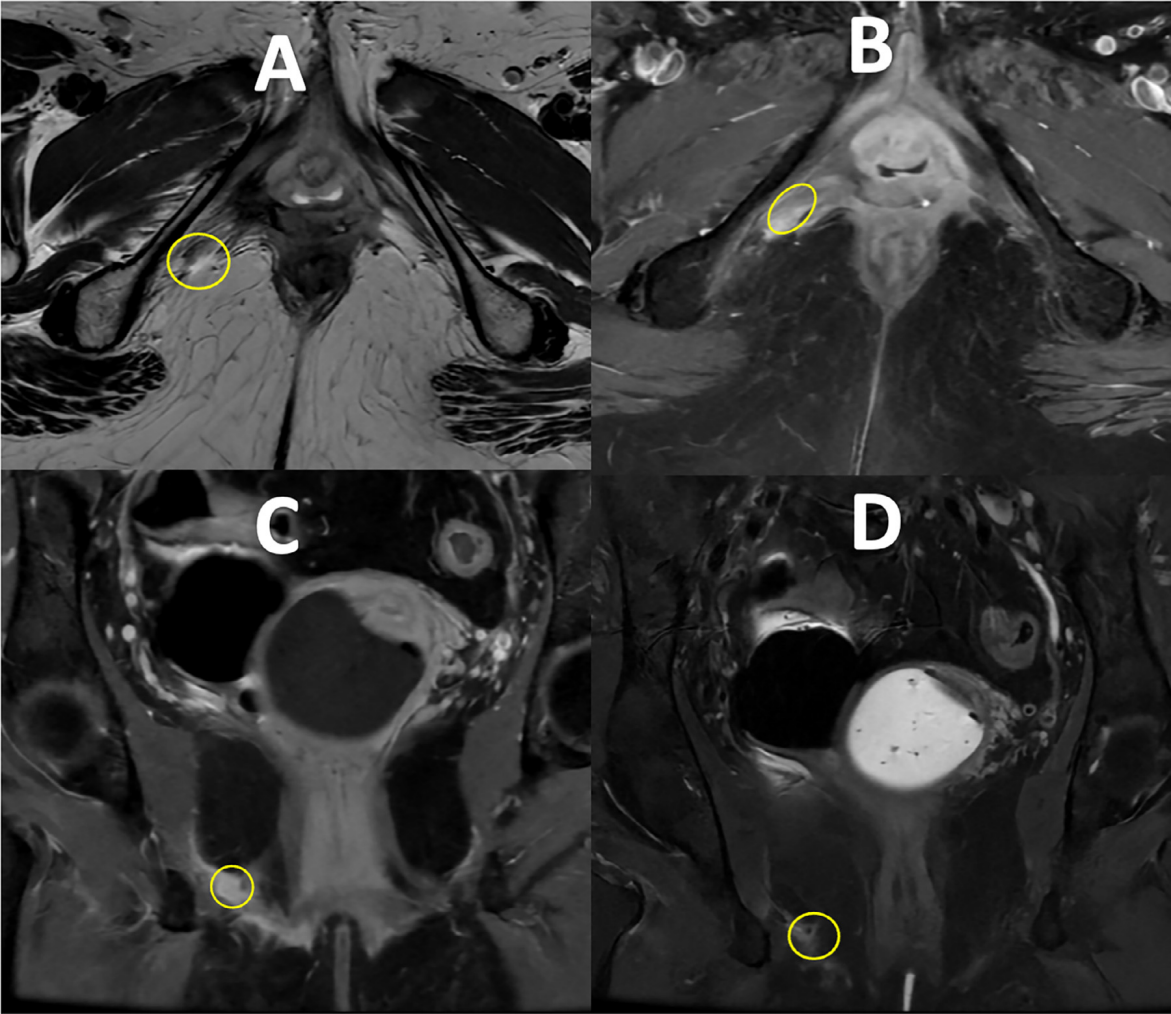
October 2024: MRI scan performed 15 months after SABR showed ongoing response of the lesion remaining isointense and no further contrast enhancement identified (A, T2w axial; B, T1w axial with contrast; C, T1w coronal with contrast; D, T2w fat-suppressed coronal).

The patient completed SABR in August 2023. A follow-up MRI performed in October 2023 showed that the treated lesion was regressed and became isointense to the muscle. Tumor board radiology review suggested this to be a nonviable fibrotic tissue, with no evidence of further active disease or pelvic lesions. On review a year later the patient remains asymptomatic from a disease progression perspective and from further radiotherapy toxicity. Comparing the imaging over a year demonstrates that SABR was successful in controlling the recurrence (Fig. 7, Fig. 8).

## Discussion

Vulval ACC is an exceptionally rare malignancy leading to unique diagnostic and therapeutic challenges. This case adds to the building repertoire of evidence base that guides the clinical teams managing patients with these unlikely diagnoses. It is vital to investigate through biopsy [1] any masses of the Bartholin’s gland in patients aged 40-years-old and older [2] for underlying ACC. ACC accounts for approximately 10-15% of all neoplasms of the Bartholin’s gland [3]. ACC in this region has a positive prognosis with good survival rates reported in both older and more recent studies. Survival corresponds to 71% at 5 years [4], 59% at 10 years [4] and still 51% at 15 years [5]. On the other hand, recurrence rates are also high with progression-free survival at 5, 10 and 15 years correlating to only 47%, 38% and 15%, respectively [5]. Given ACC’s indolent yet aggressive course, it is crucial to predict the patterns of advancing disease to guide treatment planning [[6], [7], [8]].

In the absence of formal treatment protocol, clinicians often turn to established defined pathways for malignancies pertaining to this general region. In the case of vulval ACC, management is often extrapolated from generic vulval cancer treatment algorithms [1]. For example, ESGO guidelines align with this patient's initial management where a wide local excision of the lesion was done following pelvic imaging. The guidelines then suggest, in the case of positive margins or recurrence, to consider re-excision if possible or adjuvant radiotherapy [9].

Multiple studies have demonstrated that adjuvant radiation can improve local control in cases with positive resection margins and residual tumor, respectively [10,11]. This suggests that radiotherapy can prevent local recurrence which is a major concern when the status of surgical margins is not clear. Most commonly this is delivered in the form of external beam therapy occasionally alongside brachytherapy [6]. The latter is not favored due to detrimental effect of vaginal stenosis leading to sexual inactivity as seen in this patient’s case from initial treatment.

However, there may not be as much significance in basing the adjuvant therapy plan on surgical margin status as initially described [3]. Where ACC more commonly occurs, such as in the head-and-neck exocrine glands, there is more of a consensus that negative margins can be correlated with significantly lower local recurrence rates [12]. However, the relationship between recurrence rates and resection margin status in vulval ACC is more complex. A study of 11 vulval ACC cases observed recurrences in all 4 of their patients reported to have had negative margins [4]. Another study further examined this link and found minor difference between positive (52.9%) and negative (52.1%) margins [7]. Importantly, these similar recurrence rates may be explained by adjuvant radiotherapy which was prescribed in half of the positive resection margin cases, but only in 6 out of the 27 reported in the negative margins group (22.2%). Although inconsistent follow-up periods in studies exploring the significance of margin status limits the utility of this factor for prognostic purposes, it is still generally supportive of the idea that adjuvant radiotherapy reduces recurrence rates particularly in cases of advancement into local structures and nerve tracts [13].

In this case the patient underwent WLE followed by adjuvant high-dose-rate (HDR) vaginal brachytherapy to address minimal residual disease and the positive surgical margins. This combined modality treatment achieved a disease-free interval of 4-years, underscoring its initial efficacy in managing vulvar ACC. It was only on routine surveillance imaging that recurrence was detected along the dorsal branch of the pudendal nerve. This finding adds to the growing evidence of ACC's well-documented tendency for perineural spread [7,[14], [15], [16]], even after definitive treatment, and illustrates the ongoing challenges of achieving sustained disease control in atypical anatomical sites. Despite WLE, this tumor can often persist microscopically due to the high frequency of perineural invasion [13]. This complicates adjuvant treatment decisions and often necessitates broader surgical margins and targeted radiotherapy to nerve tracts.

Perineural invasion is an independent prognostic factor and indication for adjuvant radiotherapy, albeit mostly described in the context of head-and-neck ACC [17]. The potential for perineural spread is mainly observed with positive margins [18,19] so it would be reasonable to offer perineural adjuvant therapy according to this trend. However, there is no established consensus on how far to follow nerve pathways in cases of vulvar ACC. Again, it is possible to extrapolate from head-and-neck ACC where radiotherapy is delivered to the entire grossly involved nerve [[20], [21], [22]]. More proactive cases in vulval ACC need to be seen before protocol can be established for preventing perineural spread in nonhead-and-neck sites. In contrast to vulval adenocarcinomas, which frequently metastasize to regional lymph nodes, ACC rarely involves nodal metastases, so it is generally considered unnecessary, without clinical or radiological evidence to suggest spread, to irradiate the entire pelvis [23].

The salvage therapy selected in this case was SABR which is an advanced radiation modality that delivers a more intense dose of radiation per fraction (>5 Gy) over a few fractions (1-5 fractions) than standard fractionation radiotherapy [24]. Its precision targeting allows for maximal tumoricidal effect while sparing adjacent healthy tissues and critical structures. This is particularly useful for eliminating tumors along nerve pathways and critical structures that are otherwise surgically inaccessible. The patient received a total dose of 40 Gy, administered in 5 fractions over 2 weeks. Post-treatment imaging evidenced complete radiological resolution of the lesion, reduced to residual scar tissue with no active disease on annual review. This follows the treatment protocols commonly used for invasive ACC in the head-and-neck region where SABR yields excellent local control without incurring significant morbidity [[25], [26], [27]]. This minimally invasive therapy effectively avoids the need for repeat operative procedures, and further treats patients who have already undergone HDR as in this case or earlier SABR [25,28]. Given ACC’s established good prognosis the choice of adjuvant radiotherapy must be balanced between achieving control and preservation of function. Although SABR mitigates some of these risks by minimizing surrounding tissue radiation exposure, it requires meticulous treatment planning and careful patient counselling.

The recurrence along a major nerve pathway in this case emphasizes the importance of developing therapeutic strategies that effectively address disease progression through perineural invasion. This successful use of SABR in controlling local recurrence and perineural spread adds valuable data to the limited literature on managing vulvar ACC. It reinforces SABR as a viable treatment modality, especially when surgical intervention is not feasible.

There is currently no consensus on the optimal imaging modality for surveillance of perineural spread in vulvar ACC. MRI is traditionally preferred for its high-resolution anatomical detail and superior visualization of nerve pathways, while PET-CT better detects metabolically active recurrent disease. In clinical practice, the choice is often guided by local expertise and the anatomical site of concern. Further studies are required to establish standardized follow-up protocols, particularly in nonhead-and-neck sites where perineural progression is more difficult to characterize.

By documenting these outcomes, this report contributes to the growing body of evidence guiding the management of this rare malignancy. A multidisciplinary approach that integrates surgical decisions with advanced radiotherapy techniques holds the potential to improve outcomes and expand therapeutic options for patients facing this challenging disease. Although there are no established treatment guidelines particularly in the case of locally isolated recurrent ACC, the treatment strategies should focus on either surgical re-resection or targeted radiotherapy.

## Conclusion

ACC of the vulva is a rare exocrine gland malignancy with a slowly progressive, yet relentless, course. In the case presented, ACC arose in the vulva, a highly unusual site, and its recurrence after 4 years along the dorsal branch of the pudendal nerve further underscores the complexities of managing ACC outside typical anatomic locations. The pudendal nerve was treated with salvage stereotactic ablative radiotherapy (SABR), with no evidence of active disease at 1-year follow-up. This case contributes to the limited but expanding body of literature on vulval ACC, and reinforces the importance of individualized treatment approaches, guided by multidisciplinary input, when considering the use of adjuvant radiotherapy in perineural spread of ACC.

## Patient consent

Informed consent from the patient presented in the case report has been obtained for publication.

## References

[1] Di Donato V., Casorelli A., Bardhi E., Marchetti C., Palaia I., Perniola G. (2017). Bartholin gland cancer. Crit Rev Oncol Hematol.

[2] (2014). Royal College of Obstetricians and Gynaecologists. Guidelines for the diagnosis and management of vulval carcinoma. London.

[3] Anaf V., Buxant F., Rodesch F., Simon P., van de Stadt J., Noel J.C. (1999). Adenoid cystic carcinoma of Bartholin's gland: what is the optimal approach?. Eur J Surg Oncol.

[4] Lelle R.J., Davis K.P., Roberts JA. (1994). Adenoid cystic carcinoma of the Bartholin's gland: the University of Michigan experience. Int J Gynecol Cancer.

[5] Copeland L.J., Sneige N., Gershenson D.M., Saul P.B., Stringer C.A., Seski JC. (1986). Adenoid cystic carcinoma of Bartholin gland. Obstet Gynecol.

[6] Verta S., Christmann C., Brambs CE. (2022). Adenoid cystic carcinoma of Bartholin’s gland: a case report with emphasis on surgical management. Am J Case Rep.

[7] Yang S.Y., Lee J.W., Kim W.S., Jung K.L., Lee S.J., Nam JH. (2006). Adenoid cystic carcinoma of the Bartholin's gland: report of two cases and review of the literature. Gynecol Oncol.

[8] Liu C., Roof K., Alrohaibani A., Hanley K., Dilley S. (2024). Delayed pelvic recurrence in adenoid cystic carcinoma of the Bartholin's gland. Gynecol Oncol Rep.

[9] Oonk M.H., Planchamp F., Baldwin P., Bidzinski M., Brännström M., Landoni F. (2017). European Society of Gynaecological Oncology guidelines for the management of patients with vulvar cancer. Int J Gynecol Cancer.

[10] Copeland L.J., Sneige N., Gershenson D.M. (1986). Adenoid cystic carcinoma of Bartholin gland. Obstet Gynecol.

[11] Rosenberg P., Simonsen E., Risberg B. (1989). Adenoid cystic carcinoma of Bartholin's gland: a report of five new cases treated with surgery and radiotherapy. Gynecol Oncol.

[12] Cohen A.N., Damrose E.J., Huang RY. (2004). Adenoid cystic carcinoma of the submandibular gland: a 35-year review. Otolaryngol Head Neck Surg.

[13] Segarra Vidal B., Cañete Mota S., Andrade Cadena P., Romero Ramos E., Llorca Palomera R., Illueca Carbonell J. (2021). Adenoid cystic carcinoma of the Bartholin's gland. Int J Gynecol Cancer.

[14] Zhan P., Li G., Liu B., Zhu Q., Wang Z., Wang Q. (2014). Bartholin gland carcinoma: a case report. Oncol Lett.

[15] Hsu S.T., Wang R.C., Lu C.H., Chien T.Y., Yu M.H., Wu CJ. (2013). Report of two cases of adenoid cystic carcinoma of Bartholin's gland and review of literature. Taiwan J Obstet Gynecol.

[16] Bhalwal A.B., Nick A.M., dos Reis R., Munsell M.F., Ramalingam P., Coleman R.L. (2016). Carcinoma of the Bartholin gland: a review of 33 cases. Int J Gynecol Cancer.

[17] Bernhardt D., Sterzing F., Adeberg S., Herfarth K., Katayama S., Foerster R. (2018). Bimodality treatment of patients with pelvic adenoid cystic carcinoma with photon intensity-modulated radiotherapy plus carbon ion boost: a case series. Cancer Manag Res.

[18] Mu X., Li Y., He L., Zhao C., Hua Y., Fu X. (2020). Prognostic nomogram for adenoid cystic carcinoma in different anatomic sites. Head Neck.

[19] Nakamura K., Aimono E., Tanishima S., Yamane H., Osako T., Sakashita K. (2020). Genetic profiling of patients with adenoid cystic carcinoma of the Bartholin's glands reveals potential new routes for targeted therapies: a case report. Diagn Pathol.

[20] Ellington C.L., Goodman M., Kono S.A., Grist W., Wadsworth T., Chen A.Y. (2012). Adenoid cystic carcinoma of the head and neck: a single institutional experience. Cancer.

[21] Dantas A.N., deMorais E.F., Macedo R.A., Tinôco J.M., de Morais Mda G. (2015). Clinicopathological characteristics and perineural invasion in adenoid cystic carcinoma: a systematic review. Braz J Otorhinolaryngol.

[22] Ko J.J., Siever J.E., Hao D., Simpson R., Lau HY. (2016). Adenoid cystic carcinoma of head and neck: clinical predictors of outcome from a Canadian centre. Curr Oncol.

[23] Bernstein S.G., Voet R.L., Lifshitz S., Buchsbaum HJ. (1983). Adenoid cystic carcinoma of Bartholin's gland: case report and review of the literature. Am J Obstet Gynecol.

[24] Chang JY. (2015). Stereotactic ablative radiotherapy: aim for a cure of cancer. Ann Transl Med.

[25] Nakamura Y., Umekawa M., Shinya Y., Hasegawa H., Shin M., Katano A. (2022). Stereotactic radiosurgery for skull base adenoid cystic carcinoma: a report of two cases. Surg Neurol Int.

[26] Mori Y., Kobayashi T., Kida Y., Oda K., Shibamoto Y., Yoshida J. (2005). Stereotactic radiosurgery as a salvage treatment for recurrent skull base adenoid cystic carcinoma. Stereotact Funct Neurosurg.

[27] Phan J.L., Pollard C., Wang H., Ng S.P., Sheu T., Ginsberg L.E. (2018). Dosimetric advantages of stereotactic radiosurgery as a boost to adjuvant conventional radiotherapy in the setting of adenoid cystic carcinoma of the parotid with skull base invasion. Clin Case Rep.

[28] Harris S., Chan M.D., Lovato J.F., Ellis T.L., Tatter S.B., Bourland J.D. (2012). Gamma knife stereotactic radiosurgery as salvage therapy after failure of whole-brain radiotherapy in patients with small-cell lung cancer. Int J Radiat Oncol Biol Phys.

